# Stabilization of G-quadruplex DNA structures in *Schizosaccharomyces pombe* causes single-strand DNA lesions and impedes DNA replication

**DOI:** 10.1093/nar/gkaa820

**Published:** 2020-10-12

**Authors:** Ikenna Obi, Matilda Rentoft, Vandana Singh, Jan Jamroskovic, Karam Chand, Erik Chorell, Fredrik Westerlund, Nasim Sabouri

**Affiliations:** Department of Medical Biochemistry and Biophysics, Umeå University, 901 87 Umeå, Sweden; Department of Medical Biochemistry and Biophysics, Umeå University, 901 87 Umeå, Sweden; Department of Biology and Biological Engineering, Chalmers University of Technology, 412 96 Gothenburg, Sweden; Department of Medical Biochemistry and Biophysics, Umeå University, 901 87 Umeå, Sweden; Department of Chemistry, Umeå University, 901 87 Umeå, Sweden; Department of Chemistry, Umeå University, 901 87 Umeå, Sweden; Department of Biology and Biological Engineering, Chalmers University of Technology, 412 96 Gothenburg, Sweden; Department of Medical Biochemistry and Biophysics, Umeå University, 901 87 Umeå, Sweden

## Abstract

G-quadruplex (G4) structures are stable non-canonical DNA structures that are implicated in the regulation of many cellular pathways. We show here that the G4-stabilizing compound PhenDC3 causes growth defects in *Schizosaccharomyces pombe* cells, especially during S-phase in synchronized cultures. By visualizing individual DNA molecules, we observed shorter DNA fragments of newly replicated DNA in the PhenDC3-treated cells, suggesting that PhenDC3 impedes replication fork progression. Furthermore, a novel single DNA molecule damage assay revealed increased single-strand DNA lesions in the PhenDC3-treated cells. Moreover, chromatin immunoprecipitation showed enrichment of the leading-strand DNA polymerase at sites of predicted G4 structures, suggesting that these structures impede DNA replication. We tested a subset of these sites and showed that they form G4 structures, that they stall DNA synthesis *in vitro* and that they can be resolved by the breast cancer-associated Pif1 family helicases. Our results thus suggest that G4 structures occur in *S. pombe* and that stabilized/unresolved G4 structures are obstacles for the replication machinery. The increased levels of DNA damage might further highlight the association of the human Pif1 helicase with familial breast cancer and the onset of other human diseases connected to unresolved G4 structures.

## INTRODUCTION

Nucleic acids rich in guanine bases can fold into non-canonical secondary DNA structures termed G-quadruplexes (G4s) (1). These structures are formed when four guanine residues form a planar structure—a G-tetrad—through Hoogsteen hydrogen bonds. Two or more G-tetrads can then stack on top of each other to form a G4 structure. G4 structures are stabilized by monovalent cations (e.g. K^+^ or Na^+^) that bind within the cavity between each pair of G-tetrads. G4 structures have high thermodynamic stability under physiological conditions (2), and the thermostability and likelihood of formation of a G4 structure are correlated with the length and sequence of the loop region that connects the G-tetrads (3,4). Bioinformatics analyses have revealed the enrichment of predicted G4 structures in origins of replication, gene promoters, 5′ and 3′ untranslated regions of mRNA, meiotic double-strand break hot spots, ribosomal DNA (rDNA) and telomeres (5–11). Notably, G4 structures are present in the promoters of many oncogenes, including *c-MYC*, *K-RAS* and *c-KIT*, making G4 structures potentially interesting drug targets for cancer treatment (12–14). The formation of G4 structures is important for transcriptional and translational regulation, as well as for telomere maintenance (15,16), but if they are not resolved, G4 structures can induce replication stalling and genome instability (17).

The G4-sequencing method, an *in vitro* polymerase stop assay performed in the presence of a G4-stabilizing small molecule combined with whole-genome sequencing, suggested the presence of >700 000 predicted G4 structures in the human genome (18). However, whether all of these predicted sites actually form G4 structures in the human genome needs further validation. Many studies have attempted to validate G4 formation in cells using different methods, and a common approach to demonstrate G4 formation *in vivo* is to study proteins that facilitate folding or unfolding of G4 structures. For instance, using chromatin immunoprecipitation (ChIP), nucleolin is reported to bind to G4 structures in the *c-MYC* promoter of HeLa cells (19). Also, replication protein A (RPA) and protection of telomeres 1 (Pot1) have been reported to interact with distinct telomeric G4 DNA using single-molecule Förster resonance energy transfer (20). ChIP has been used to show that members of the evolutionarily conserved Pif1 5′–3′ family of helicases are enriched at predicted G4 structures in *Saccharomyces cerevisiae* and *Schizosaccharomyces pombe* and are needed to prevent replication arrest and genome instability at these sites (8,21,22). The human PIF1 helicase (hPIF1) is associated with familial breast cancer (23), and in these families a point mutation is found in the Pif1-encoding gene (23). The mutation is located in the Pif1 signature motif (24), which is a conserved 23 amino acid region in Pif1 helicases (25,26), and *S. pombe* cells carrying the corresponding mutation are inviable (23). *In vitro*, Pif1 helicases from many organisms, including humans, *S. pombe*, *S. cerevisiae* and bacterial Pif1 family homologs, bind and unwind G4 structures (21,25,27–32). However, recombinant Pfh1, the Pif1 homolog in *S. pombe*, which carries the corresponding breast cancer-associated mutation loses its G4-unwinding activity, suggesting that this conserved region is important for resolving G4 structures (25).

**Figure 1.**
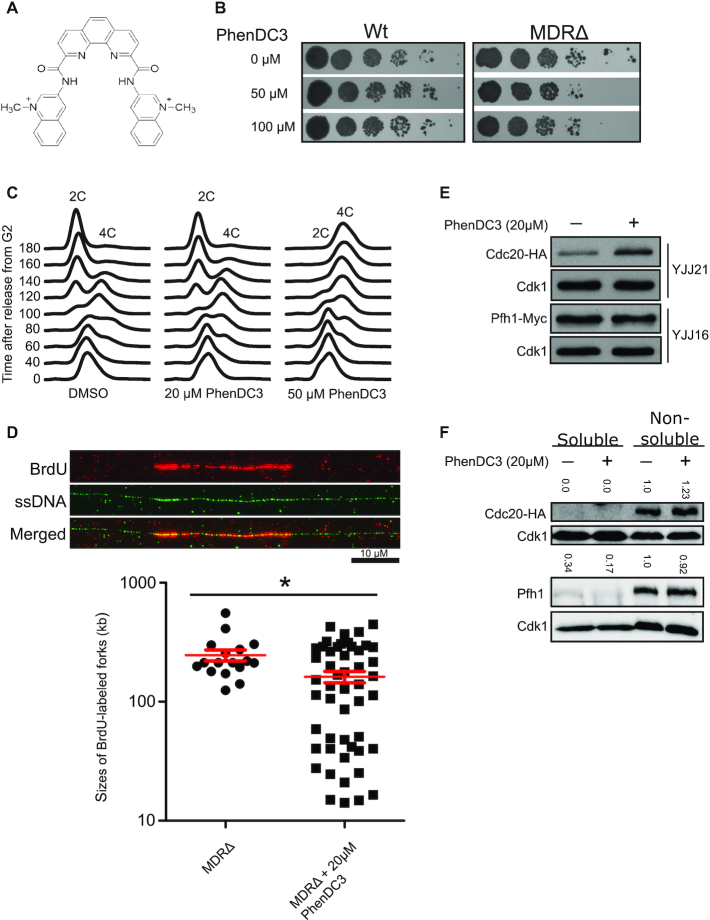
Stabilized G4 structures perturb DNA replication. (**A**) The structure of PhenDC3. (**B**) Serial dilution spot assay of wt (YNS112) and *MDRΔ* (YNS219) cells in the presence of 0 μM (DMSO), 50 μM or 100 μM PhenDC3. (**C**) G2-arrested YJJ32 (*cdc25–22 MDRΔ cdc20-HA*) cells were synchronized and released in the presence of 0 μM (DMSO), 20 μM or 50 μM PhenDC3. Samples were taken at the indicated times (min). The experiment was repeated at least three times for each condition, and representative flow cytometry profiles are shown. (**D**) DNA fiber analysis to monitor DNA replication of *MDRΔ cells* in the presence of 0 μM (DMSO) or 20 μM PhenDC3. G2-arrested YIO4 (*MDRΔ cdc25–22*, *hENT1* and *hsv‐tk*) cells were released at the permissive temperature of 25°C in the presence of BrdU. The images show a DNA fiber stained for BrdU (top, red), ssDNA (middle, green) and merged (bottom). The graph shows the measured sizes of the BrdU-labeled DNA fibers. For the untreated *MDRΔ* cells 17 fibers were counted, and for the PhenDC3-treated *MDRΔ* cells 50 fibers were counted. The experiment was repeated twice, and data from a representative experiment are shown. The mean size of the BrdU-labeled fibers is shown in red, and error bars represent the standard error of the mean. * *P* < 0.05 according to the Mann–Whitney U-test. (**E** and **F**) Western blot analysis of the total cellular (E) or cytoplasmic/chromatin (F) Cdc20 and Pfh1 levels in untreated and PhenDC3-treated cells (20 μM). Total cellular, cytoplasmic and chromatin proteins were extracted and separated on a 9% SDS-polyacrylamide gel. Cdk1 was used as the loading control. The numbers at the top of each gel indicate the relative amounts of Cdc20 or Pfh1 in each band normalized with their corresponding Cdk1 bands and compared to the normalized Cdc20 or Pfh1 bands from the non-soluble fraction of the sample without PhenDC3 treatment.

Another approach used to study the biological roles of G4 structures is the use of small molecules designed to specifically recognize and/or stabilize G4 structures (33). For instance pyridostatin, a small G4-stabilizing molecule, inhibits telomerase activity *in vitro* (34), slows replication fork progression (35) and elevates the levels of γ-H2AX, a marker of DNA damage, in human cells (36). Also, pyridostatin conjugated to a fluorescent molecule co-localizes with human PIF1 in human cells, indicating that hPIF1 is needed at those sites for resolving the stabilized G4 structures (36). The use of G4 ligands relies heavily on their high affinity for G4 structures over other DNA structures, especially duplex DNA, and on their uptake into cells. The bisquinolinium compound PhenDC3 (Figure 1A) is a well-recognized and commonly used G4 ligand (37–39), and PhenDC3 displays exceptional affinity and selectivity for G4 structures and telomeric regions of human chromosomes (39). In *S. cerevisiae*, the presence of PhenDC3 enhances genome instability in cells carrying human CEB1 mini satellites that encompass tandem repeats of G4 sequences (40,41).

PhenDC3 has also been successfully used to study the formation of G4 structures *in vitro* in *S. pombe* and *S. cerevisiae* genomic DNA (42); however, the type of lesions that PhenDC3 may induce has not been studied. The base excision repair (BER) pathway repairs base lesions caused by for instance oxidation, alkylation and deamination. Recently, a BER-based single-molecule imaging technique was used to identify and quantify ionizing radiation-induced single-stranded DNA (ssDNA) lesions (43). This technique identifies different types of DNA damages caused by DNA damaging agents based on proficiency of a particular enzyme in a BER enzyme mixture (43,44).

Bioinformatics analyses suggest that there are 446 G4 structures dispersed throughout the *S. pombe* genome (8), and here we used *S. pombe* to explore how stabilized G4 structures affect DNA replication. *S. pombe*, which has been referred to as a ‘micro-mammal’ (45), is an excellent model organism that has provided enormous insights into cellular mechanisms. However, *S. pombe* cells have rarely been used to study G4 formation, despite the fact that similar genomic features overlap between G4 motifs in *S. pombe* and other model species, such as mice and *S. cerevisiae* (5,6,46,47). Also, *S. pombe* cells have not been extensively used for chemical biology experiments due to their efficient efflux pumps, which extrudes toxic substances from the cell. By deleting the two ABC transporter proteins, Pmd1 and Bfr1, the Kapoor lab was able to engineer an *S. pombe* strain that is sensitive to many drugs (48); however, G4-stabilizing compounds were not tested in their study.

In our present study, we show that the same *S. pombe* strain is sensitive to PhenDC3. Furthermore, in the presence of PhenDC3 and at single-molecule resolution, we detected reduced tract length of newly replicated DNA and increased numbers of ssDNA lesions, suggesting that these cells have replication defects. We also examined whether PhenDC3 can be used as a tool in ChIP combined with sequencing (ChIP-seq) experiments to determine the presence of G4 structures *in vivo*. Results from our ChIP-seq experiments performed at the optimal PhenDC3 concentration, where we used the catalytic subunit of the leading-strand DNA polymerase as a marker for slowed replication, identified genomic G4 sites that could impede replication fork progression. *In vitro* G4 assays confirmed that these sequences form G4 structures in the presence of KCl and that these predicted G4 structures cause stalling of DNA synthesis. In particular, the use of PhenDC3 improved our detection of G4 structures that are less stable. Furthermore, recombinant Pfh1 (49) efficiently unwound these intramolecular G4 structures. The combination of these different methods strongly suggests that stable/stabilized G4 structures form replication barriers in *S. pombe* and that Pfh1 is needed during replication of these sites to prevent the formation of DNA breaks.

## MATERIALS AND METHODS

### Strains

The yeast strains used in this study are listed in Supplementary Table S1.

### Oligonucleotides

The oligonucleotides used in this study are shown in Supplementary Table S2.

### PhenDC3 sensitivity assay

PhenDC3 was synthesized as previously described (42). To test *S. pombe* sensitivity to PhenDC3, YNS112 (wild-type) and YNS219 (*MDRΔ*) strains were grown overnight in liquid EMM2 media (Formedium). Starting with 2.4 × 10^6^ cells/ml, 5-fold serial dilutions of the cells were made, and 5 μl of these dilutions were spotted on EMM2 agar plates containing 1.3% (v/v) DMSO or 50 or 100 μM PhenDC3. Plates were incubated at 30°C for 5 days. The determination of the optimal PhenDC3 concentration was performed by growing YJJ21 cells in liquid EMM2 media containing 0.03% (v/v) DMSO and 10, 20, 50 or 100 μM PhenDC3. Five-fold serial dilutions of the cells were spotted on EMM2 plates, and these were incubated at 30°C for 5 days. Each experiment was performed in triplicate. To determine the doubling time, exponentially growing YJJ21 cells were inoculated at 1 million cells/ml in EMM2 media containing 0.13% DMSO (v/v), 20 or 50 μM PhenDC3. Cultures were grown at 30°C at 180 rpm and counted after 12 h using a Bürker chamber. Next, the cells were diluted to 1 million cells/ml in the presence of fresh DMSO, or 20 or 50 μM PhenDC3, grown for additional 12 h, and counted to calculate the doubling time. Three independent experiments were performed to calculate the doubling time using the following formula: Doubling time = *t* / log_2_ (*x* /*x*_0_), where *t* is time in hours; *x* is counted cell number at 12 h and *x*_0_ is counted cell number at 0 h.

### DNA fiber analysis

*S. pombe* cells (YIO4) were grown to 10^7^ cells/ml in the presence of 20 μM PhenDC3 or 0.03% (v/v) DMSO at 25°C in liquid EMM2 media. Subsequently, the cultures were diluted twice and incubated at 36.5°C for 5 h to arrest the cells at the G2-phase. These cells were then released from the G2-phase by shifting the temperature to 25°C. At 30 min after release from the G2-phase, 66 μM final concentration of bromodeoxyuridine (BrdU) was added and the cells were allowed to incorporate the BrdU into their DNA for 35 min. A stop solution (250 mM EDTA (pH 8.0) and 0.16% sodium azide) was added, and cells were harvested by centrifugation and resuspended in cold 70% ethanol. The ethanol was removed by centrifugation, and the cells were washed in PBS and resuspended in digestion buffer (1 M sorbitol, 1 mM EDTA, and 10 mM Tris-HCl (pH 7.0)) for cell-wall digestion using 200 U/ml lyticase from *Arthrobacter luteus* (Sigma-Aldrich). Cells were pelleted and resuspended in 100 μl PBS. Spreading of DNA fibers on glass slides was performed as previously described (50). Briefly, 8 μl of cell suspension was dropped at one end of a microscope slide (Superfrost Plus, Thermo Fisher Scientific) and allowed to partially dry. After lysing the cells with 30 μl lysis solution (50 mM Tris-HCl (pH 7.4), 25 mM EDTA, 500 mM NaCl, 0.1% Nonidet *P*-40, 1% sodium dodecyl sulfate (SDS), and 5 mM β-mercaptoethanol), their DNA was stretched along the slide by tilting the slide to 15° and fixing with 4% paraformaldehyde. The slides were incubated in 0.5 M NaOH for 25 min to denature the DNA. Immunostaining of BrdU incorporated into DNA was detected using rat anti-BrdU clone [BU1/75 (ICRI)] primary antibody (ABD Serotec) and goat anti-rat IgG Alexa Fluor 568 secondary antibody (Life Technologies), while ssDNA was detected using anti-DNA antibody, single-stranded, clone 16–19 primary antibody (Sigma Aldrich) and goat anti-mouse IgG2a (γ2a) Alexa Fluor 488 secondary antibody (Life Technologies). An Axio Imager Z1 microscope (Zeiss) was used to visualize the stained DNA fibers, and images of untangled DNA fibers were obtained at randomly selected fields of view. Only DNA fibers with BrdU labeling at intact ssDNA ends or DNA fibers with BrdU labels measuring >70 μm were selected for analysis using the Zen 2.6 blue edition (Zeiss) and ImageJ software packages. The experiments were repeated two times independently, and at least 17 images were taken for each condition in each experiment. DNA fibers were measured in micrometers and converted to kilobases using a conversion factor of 1 μm BrdU label corresponding to roughly 2 kb (51).

### Protein extraction and Western blot

An alkaline extraction method described in (52) was used to extract proteins from *S. pombe* cells. A total of 10 ml YJJ21 or YJJ16 cells were grown for 12 h in the presence of 20 μM PhenDC3 or 0.03% (v/v) DMSO. Cells were pelleted, washed with 1 ml distilled water, resuspended in 0.3 ml distilled water, and 0.3 ml 0.6 M NaOH was added and the cells were incubated for 10 min. After centrifugation, the supernatant was carefully removed and the cells were gently resuspended in 70 μl 2× sample buffer (100 mM Tris-HCl (pH 6.8), 4% (w/v) SDS, 0.2% (w/v) bromophenol blue and 200 mM β-mercaptoethanol). Proteins were separated on a 9% SDS-polyacrylamide gel and blotted onto a PVDF membrane. Cdc20-HA and Cdk1 were detected using anti-HA (Santa Cruz Biotechnology) and anti-Cdk1 (Abcam) antibodies, respectively. Pfh1–13Myc in YJJ16 cells was detected using anti-Myc (Takara Bio), while Pfh1 in YJJ21 cells was detected with anti-Pfh1. For the cellular fractionation experiments, the *S. pombe* strain (YJJ21) was grown in liquid EMM2 media for 12 h in the presence of 20 μM PhenDC3 or 0.03% (v/v) DMSO. Harvested cells were resuspended in ChIP lysis buffer (50 mM Hepes/KOH (pH 7.5), 140 mM NaCl, 1 mM EDTA, 1% Triton X-100, and 0.1% Na-deoxycholate) and lysed using glass beads in a FastPrep-24™ homogenizer (MP Biomedicals). Lysed cells were separated into the soluble fraction (supernatant) and insoluble fraction (pellet) by centrifugation at 20,900 × ***g*** for 15 min at 4°C. Fractions were treated with SDS sample buffer prior to SDS-PAGE and Western blot analysis.

### Flow cytometry analysis

YJJ32 *S. pombe* cells were grown in liquid PMG medium (Formedium) at 25°C. Cells were diluted to 1.5 × 10^6^ cells/ml and grown for 30 min at 25°C prior to arresting the cells at late G2-phase by incubating for 5 h at 36.5°C. After 5 h, the cells were rapidly cooled down to 25°C in an ice bath to synchronously release them from the late G2-phase. The cultures were divided into different flasks and supplemented with 0.05% DMSO, or 20 or 50 μM PhenDC3. Samples for flow cytometry analysis were taken immediately after the cells were cooled down to 25°C (time 0), after 40 min, and then every 20 min until 240 min. For cell fixation, 2 ml of cell suspension (approximately 3 × 10^6^ cells) was transferred into a 15 ml tube with 1 ml of stop solution (50 mM EDTA and 0.1% sodium azide) and incubated for 10 min. After centrifugation at 1000 × ***g*** for 5 min, the cells were resuspended in 700 μl of 70% EtOH and stored at 4°C. A total volume of 300 μl cells (1.5 × 10^6^ cells) was washed in 3 ml of 50 mM sodium citrate (pH 8) and incubated overnight in 500 μl of 50 mM sodium citrate (pH 8) and 0.2 mg/ml of RNAseA (Thermo Fisher Scientific). The cells were then separated by sonication and mixed with 500 μl of staining solution (50 mM sodium citrate (pH 8) and 1 × Sybr Green I (Thermo Fisher Scientific)). The cells were incubated at room temperature for at least 30 min before analysis on a Beckman Coulter Cytomics FC500 flow cytometer. Data were smoothed in OriginLab software for better visualization.

### ChIP-seq

Logarithmically growing *S. pombe* cells (strain YJJ21) were grown in liquid EMM2 media for 12 h in the presence of 20 μM PhenDC3 or 0.03% (v/v) DMSO. Subsequent procedures for the ChIP experiments were performed as previously described (8). Briefly, cells were crosslinked with 1% formaldehyde for 5 min at room temperature. The cell-walls of the crosslinked cells were then disrupted in a FastPrep-24™ benchtop homogenizer. Chromatin was isolated and sheared to lengths of approximately 300 bp using a Covaris E220, and Cdc20-HA was immunoprecipitated with anti-HA (Santa Cruz Biotechnology). Both immunoprecipitated and input DNA were purified, and 10 ng DNA was used to prepare sequencing libraries using the NEBNext Ultra DNA Library Prep Kit (New England Biolabs). Indexed libraries were sequenced using the Illumina Hiseq sequencing platform (Novogene), and two biological replicates were sequenced for each condition.

### ChIP-seq data analysis

ChIP-seq data were trimmed using Trim Galore (v.0.4.1) (REF: https://github.com/FelixKrueger/TrimGalore), and the quality was assessed using FastQC (v.0.11.5) (REF: https://github.com/s-andrews/FastQC). Reads were mapped against the *S. pombe* reference genome ASM294v2 by Bowtie2 v.2.3.2 (53), and reads with a mapping quality below 10 or with a 4 in the flag field were filtered out using SAMtools v.1.5 (54) in order to keep only uniquely mapped reads. Peaks were detected using MACS2 v.2.1.0 (55) and https://github.com/taoliu/MACS/. The IDR (irreproducible discovery rate) pipeline from ENCODE was used to generate high-confidence peak lists from each pair of biological replicates, as well as to evaluate replicate reproducibility (56). An uncorrected *P*-value cutoff of 0.001 was used in the MACS2 analysis to generate both the low-confidence and high-confidence peaks needed in the IDR analysis. Each replicate pair was then merged into a single set of reproducible peaks (*N*_t_) using IDR (IDR < 0.01). To evaluate reproducibility, reads from each pair of replicates were merged and randomly subsampled into two pseudo replicates (pRep) using SAMtools. Reproducible peaks for the pseudo replicates (*N*_p_) were again detected using MACS2 and IDR and compared to the number of peaks in the original replicates (*N*_p_/*N*_t_). An *N*_p_ within a factor of 2 from *N*_t_ (*N*_p_/*N*_t_ <|2|) indicates reliable replicates according to ENCODE, and this condition was fulfilled for all of our replicates (Table 1, Supporting File 1). The sequencing data have been deposited at the European Nucleotide Archive (ENA, www.ebi.ac.uk/ena) under accession number PRJEB37862.

**Table 1.** Number of peaks detected in ChIP-seq samples

Sample	Pearson correlation	MACS2 Rep1;Rep2	IDR (*N*_t_)	MACS2 pRep1;pRep2	IDR pRep (*N*_p_)	*N*_p_/*N*_t_
mock	0.97	1654;1567	344	1590;1469	527	1.53
treated	0.99	1657;1599	568	1405;1183	538	0.95

pRep; pseudoreplicates

### DNA primer extension assay

The DNA primer extension assay was performed as described previously (57). Briefly, TET-labeled primer (1 μM) was annealed to 1.25 μM oligonucleotides containing G4 or non-G4 (*S. pombe ade6^+^*) sequences. Extension of the annealed primer was performed at 37°C for 1 min in the presence of either 1% (v/v) DMSO and 100 mM KCl or 0.1 μM PhenDC3 and 100 mM KCl using 0.063 U of an exonuclease-deficient Klenow fragment of *Escherichia coli* DNA polymerase (Thermo Fisher Scientific). Reaction products were separated on 10% polyacrylamide gels containing 8 M urea, 25% formamide, and 1 × Tris/borate/ethylenediaminetetraacetic acid (TBE). Bands were visualized and quantified using a Typhoon Scanner 9400 (GE Healthcare) and the ImageQuant 5.2 software (GE Healthcare).

### qPCR stop assay

The qPCR stop assay was performed as described previously (42). Briefly, each qPCR was carried out in a volume of 10 μl containing 0.3 μM primer pair, 33 ng *S. pombe* genomic DNA, and 1 × SyGreen mix (PCR Biosystems) in the presence of either 0.65% (v/v) DMSO, 25 mM KCl, or 25 mM KCl and 0.5 μM PhenDC3. The reactions were performed on a LightCycler 96 thermocycler (Roche) using the following program: 95°C for 5 min (1 cycle) followed by a 2-step reaction of 85°C for 10 s and 60°C for 20 s (33 cycles) in one-point acquisition mode. ΔΔ*C*_q_ values were determined to express the relative DNA amplification.

### CD measurements

G4 oligonucleotides (50 μM) in water or buffer (10 mM Tris-HCl pH 7.5, 100 mM KCl) were heated at 95°C for 5 min and allowed to fold at room temperature for 3 h, and 5 μM folded G4 oligonucleotides were incubated with 10 μM PhenDC3 or 1.3% (v/v) DMSO. CD spectra were recorded between 205 and 350 nm at 25°C using a JASCO-720 spectrometer with a Peltier temperature control in a quartz cuvette (0.1 cm path length). Each spectrum was the accumulation of four measurements. A buffer (10 mM Tris-HCl (pH 7.5) and 100 mM KCl) containing 1.25% (v/v) DMSO or 10 μM PhenDC3 was used as a blank for baseline corrections. All data were normalized to molar ellipticity by using the formula below:

$$\begin{equation*}\ \left[ {\rm{\theta }} \right] = \frac{{m^\circ \ \times M}}{{10\ \times L \times C}}\ \end{equation*}$$

Where *m°* is CD signal in millidegrees; *M* is molecular weight of oligonucleotides in g/mol; *L* is path length of cell in cm; *C* is concentration of oligonucleotides in g/l.

### Labeling and folding of G4 oligonucleotides

T4 polynucleotide kinase (PNK) (Thermo Fisher Scientific) and γ-^32^P-ATP were used to 5′ end label 0.5 μM 10A-G4 oligonucleotides or their mutated variants at 37°C for 75 min. Subsequently, 1 μl 0.5 M ethylenediaminetetraacetic acid (EDTA) was added and the reaction was incubated at 78°C for 1 min to inactivate the T4 PNK. Labeled DNA was purified on a G50 column (GE Healthcare).

To fold the G4 oligonucleotides, 20 μl of labeled 0.2 μM 10A-G4 oligonucleotides, or their mutated variants, was mixed with an equal volume 2 × folding buffer (20 mM Tris (pH 7.5) and 200 mM KCl). The reaction was incubated at 95°C for 5 min and allowed to cool down to room temperature for 3 h. The oligonucleotides were loaded on a 10% native polyacrylamide gel containing 50 mM KCl and separated in a cold ice box run at 100 V for 80 min.

### Helicase and helicase trap assays

The Pfh1 G4 unwinding assay was performed as reported previously (27). Briefly, 1 nM folded 10A-G4 oligonucleotides in a reaction mixture containing 50 mM Tris-HCl (pH 8.5), 2 mM 1,4-dithiothreithol (DTT), 2 mM ATP, 0.25 mg/ml bovine serum albumin (BSA), 2 mM MgCl_2_ and 100 mM KCl were incubated for 10 min at 30°C with various concentrations of Pfh1 (0, 0.09, 0.9, 9 and 18 nM). For the helicase trap assay, 10 nM oligonucleotide complementary to the sequence of the G4 motifs was included in the reaction. The reaction was stopped by adding 4 μl 6 × stop solution (60 mM EDTA (pH 8.0), 40% (w/v) sucrose, 0.6% SDS, 0.2% bromophenol blue, and 0.5 mg/ml proteinase K) on ice. Reaction products were separated by electrophoresis using a 10% polyacrylamide gel containing 50 mM KCl. Bands were visualized and quantified using a Typhoon Scanner 9400 and ImageQuant 5.2 software. The amount of unwound DNA was calculated using the formula [% unwound = 100 × (*P*/(*P* + *S*))], where *P* is the pixel intensity of unwound DNA band and S is the background minus pixel intensity of the corrected intact DNA band.

### Single molecule imaging of PhenDC3-induced DNA damage

YJJ21 *S. pombe* cells were grown in 50 ml EMM2 media containing 0.066% (v/v) DMSO or 20 or 50 μM PhenDC3 for 16 h until reaching 10 million cells/ml. Genomic DNA was isolated using CHEF Genomic DNA Plug Kits (Bio-Rad) following the manufacturer’s protocol.

Agarose plugs containing DNA isolated from either untreated or PhenDC3-treated cells were washed in 500 μl of Milli-Q for 1 h at room temperature with gentle shaking. The supernatant was discarded, and each plug was resuspended in 200 μl of Milli-Q. The agarose plugs were first melted at 55°C for 5 min and then incubated at 42°C for 5 min. After thermal equilibration of the plugs, 1 U of agarase (Thermo Fisher Scientific) was added and the samples were incubated at 42°C for 1 h. The agarase-digested plugs were stored at 4°C until further use. Before the labeling of DNA damage, the samples were heated at 55°C for 5 min.

DNA samples (100 ng) were incubated with 2.5 U each of apurinic/apyrimidinic endonuclease 1 (APE1), formamidopyrimidine glycosylase (FpG), Endonuclease III (Endo III), endonuclease IV (Endo IV), and endonuclease VIII (Endo VIII) in 1× CutSmart Buffer (New England BioLabs (NEB)) for 1 h at 37°C. These enzymes are collectively referred to as the ‘enzyme cocktail’. All enzymes were procured from NEB. The PhenDC3 damage sites were labeled with dNTPs (Sigma-Aldrich) (1 μM of dATP, dGTP, dCTP, and 0.25 μM dTTP and 0.25 μM Aminoallyl-dUTP-ATTO-647N (Jena Bioscience)) and DNA polymerase 1 (DNA pol 1) (1.25 U) at 20°C for 1 h in 1 × NEBuffer 2 (NEB). Finally, the reaction was terminated with 2.5 μl of 0.25 M EDTA (Sigma-Aldrich) and the samples were stored at 4°C until analysis.

A total of 5 μl of the fluorescently labeled DNA was stained with 320 nM YOYO-1 (Invitrogen) in a final volume of 50 μl of 0.5 × TBE, and the samples were heated at 55°C for 15 min. One microliter of β-mercaptoethanol (Sigma-Aldrich) was added to the samples just before stretching to avoid photo bleaching of the samples while imaging. A total of 3.5 μl of each sample was then stretched on the silanized coverslips by placing the labeled DNA samples at the interface of the coverslip and a glass slide (Thermo Scientific, Menzel-Gläser) (58). No. 1 coverslips (22 × 22 mm, MARIENFELD Laboratory Glassware) were submerged in a mixture of 1% (3-aminopropyl) triethoxysilane (Sigma-Aldrich), 1% allyltrimethoxysilane (Sigma-Aldrich) and acetone overnight. The silane-coated coverslips were rinsed with an acetone:water solution (2:1 v/v) to remove any residues and were dried by air purging. The air-dried coverslips were stored in a parafilm tight petridish at room temperature and utilized within a week.

The DNA molecules were stretched on the silanized coverslips and then imaged with a fluorescence microscope (Zeiss Observer.Z1) equipped with an Andor iXON Ultra EMCCD camera and a Colibri 7 LED illumination system. This set up has band-pass excitation filters (475/40 and 640/30) and bandpass emission filters (530/50 and 690/50) for YOYO-1 and Aminoallyl-dUTP-ATTO-647N, respectively. An EM gain setting of 100 and exposure times of 30 and 500 ms for YOYO-1 and Aminoallyl-dUTP-ATTO-647N, respectively, were used.

A custom-made software that reports the damage as dots/μm was used to analyze the data. The data was then converted to dots/MBp using a conversion factor of 3000 bp/μm. The damage caused by PhenDC3 is reported in terms of damage detected (DD), which is the total number of sites detected by the enzyme cocktail.

## RESULTS

### PhenDC3 impairs the growth of MDR-deleted *S. pombe* cells

Ligands that stabilize G4 structures are powerful tools to study G4 structure formation (33,59,60); however, a majority of the studies to identify G4-forming sequences with the aid of G4 ligands have so far been performed *in vitro*. Wild-type (wt) *S. pombe* cells are resistant to multiple drugs due to efficient efflux pumps that expel toxins out of the cell (48), and thus *S. pombe* cells have not been extensively used in drug discovery. Therefore, to perform these experiments we utilized a strain with two ABC transporters deleted, *brf1^+^* and *pmd1^+^*, hereafter referred to as the ‘multiple drug resistant-deleted’ (*MDRΔ*) strain. This strain is sensitive to a number of drugs (48), including a new quinazoline-based G4 stabilizer (61). We performed spot dilution assays and compared the growth of wt and *MDRΔ* cells in the presence of DMSO and in the presence of 50 or 100 μM PhenDC3 (Figure 1B). At these concentrations of PhenDC3, the growth of wt cells was unaffected while the growth of *MDRΔ* cells was impaired (Figure 1B), indicating that PhenDC3 was taken up by the *MDRΔ* cells and inhibited cell growth, and thus that these cells could be used for further experiments.

### Prolonged S-phase and replication defects in PhenDC3-treated cells

To determine the impact of PhenDC3 on *S. pombe* cell cycle progression in synchronized cells, we mated the temperature-sensitive *cdc25–22* strain with the *MDRΔ* strain and generated the *cdc25–22 MDRΔ* (YJJ32) strain. At the restrictive temperature (36.5°C), *cdc25–22* cells arrest at the G2-phase, and can then be released at the permissive temperature (25°C) and followed during the cell cycle in a synchronous manner. We therefore arrested the *cdc25–22 MDRΔ* cells at 36.5°C, released them in the presence of DMSO or 20 or 50 μM PhenDC3 at 25°C, and collected samples every 20 min for up to 3 h. Flow cytometry was used to monitor the cell cycle phase of these samples. While the cells treated with DMSO or 20 μM PhenDC3 entered S-phase at about 60 min after release and completed the cell cycle by returning to G2-phase after 180 min, the 50 μM PhenDC3-treated cells showed a slightly delayed and significantly prolonged S-phase, which was still not completed even at 180 min after release (Figure 1C), consistent with the observed growth defect at this PhenDC3 concentration (Figure 1B). These data suggest that the observed growth defect and prolonged S-phase detected in the presence of PhenDC3 are due to replication progression defects.

To investigate whether DNA replication is affected by PhenDC3 treatment, we performed a DNA fiber analysis using an *S. pombe* (YIO4) strain with the *MDRΔ cdc25–22*, *hENT1* and *hsv‐tk* background that was engineered to incorporate extracellular thymidine or its analogues into DNA through the nucleotide salvage pathway. These cells were grown in the presence of DMSO or 20 μM PhenDC3, arrested at G2-phase, and released at the permissive temperature. At 30 min after release, the thymidine analogue BrdU was added and allowed to be incorporated into the newly replicated DNA during early S-phase. The DNA from these cells was spread on microscope slides, immunostained with anti-BrdU and anti-ssDNA antibodies, and visualized by fluorescence microscopy. Cells treated with 20 μM PhenDC3 showed significantly reduced lengths of newly replicated DNA compared to cells without PhenDC3 treatment (Figure 1D), showing that DNA replication is impaired in these cells. Moreover, the proportion of shorter BrdU labels in the DNA was increased in these cells (Supplementary Figure S1). However, because we neither detected prolonged S-phase by flow cytometry analysis nor growth defects at 20 μM PhenDC3, our data suggest that more origins could be fired to complete S-phase without any delays. In fact, we consistently detected more BrdU-labeled DNA fibers in the PhenDC3-treated cells than the DMSO-treated control cells (Supplementary Table S3), supporting the above argument. The leading-strand DNA polymerase ϵ (Polϵ) is needed for replication origin firing (62). To determine if more origins were fired in PhenDC3-treated cells, we performed Western blot analysis to examine the protein levels of Pol2/Cdc20, the catalytic subunit of Polϵ. Indeed, we found higher levels of Cdc20 in PhenDC3-treated cells compared to untreated cells (Figure 1E), suggesting that there was an increase in origin firing. Furthermore, the increased protein levels of Cdc20 found in the PhenDC3-treated cells was only detected in the non-soluble protein fractions (Figure 1F). These data suggest that PhenDC3-treated cells have higher levels of chromatin-bound Cdc20 than untreated cells. In contrast, we did not detect higher levels of the replisome component Pfh1 in PhenDC3-treated cells (Figure 1E and F), suggesting that the increased levels of Cdc20 might be due to increased origin firing rather than stalled replication forks.

### Identification of Cdc20-associated G4 motifs in the *S. pombe* genome

Next, we wanted to determine if the reduced levels of DNA synthesis in the PhenDC3-treated cells were due to more strongly stabilized G4 structures in the genome. In previous studies, we used ChIP to pull down Cdc20 in order to monitor replication fork progression and demonstrated that sites that have slowed replication movement have enhanced Cdc20 binding (5,63). Using the algorithm (G_≥3_ N_1–25_)3 G_≥3_ (5), where G represents a guanine base and N represents loop regions of not >25 nucleotides, a bioinformatics search for DNA sequences containing predicted G4 structures, so-called G4 motifs, identified 446 predicted intramolecular G4 structures in the *S. pombe* genome (8). This number excludes G4 motifs within repetitive telomeric DNA and rDNA, because a majority of these sequences are not included in the assembled *S. pombe* genome (64). About 10% of the G4 motifs in *S. pombe* have enhanced Cdc20 binding (8). We therefore anticipated that PhenDC3-stabilized G4 structures would induce increased Cdc20 occupancy, resulting in the detection of less stable/more quickly resolved G4 structures that are not otherwise detected in untreated samples and/or resulting in enhanced stabilization of already detected G4 structures that resulted in even higher occupancy by Cdc20 in PhenDC3-treated cells.

To perform ChIP with Cdc20, we created a yeast strain (YJJ21) expressing Cdc20-HA (46) in the *MDRΔ* background. We tested the minimal PhenDC3 concentration for the optimal growth of this strain, and similar to the *MDRΔ* parent strain (YNS219) growth of *Cdc20-HA MDRΔ* was impaired at 50 and 100 μM PhenDC3, while PhenDC3 concentrations of 20 μM or lower showed no adverse effect on growth (Supplementary Figure S2A). Furthermore, while the doubling times for cells treated with 20 μM PhenDC3 (162 min) were unaffected compared to the untreated cells (168 min), the doubling time for cells treated with 50 μM PhenDC3 increased significantly to 185 min (Supplementary Figure S2B, *P* = 0.002). Based on these experiments, we grew the strain in the presence of 20 μM PhenDC3 (to reduce the possibility of secondary defects of PhenDC3-treatment) or DMSO (as the control) for the ChIP-seq experiments and then immunoprecipitated the DNA associated with Cdc20 and performed whole-genome sequencing. The input DNA used for ChIP was also sequenced and used as a control.

### Bioinformatics analysis of the ChIP-seq experiments

**Figure 2.**
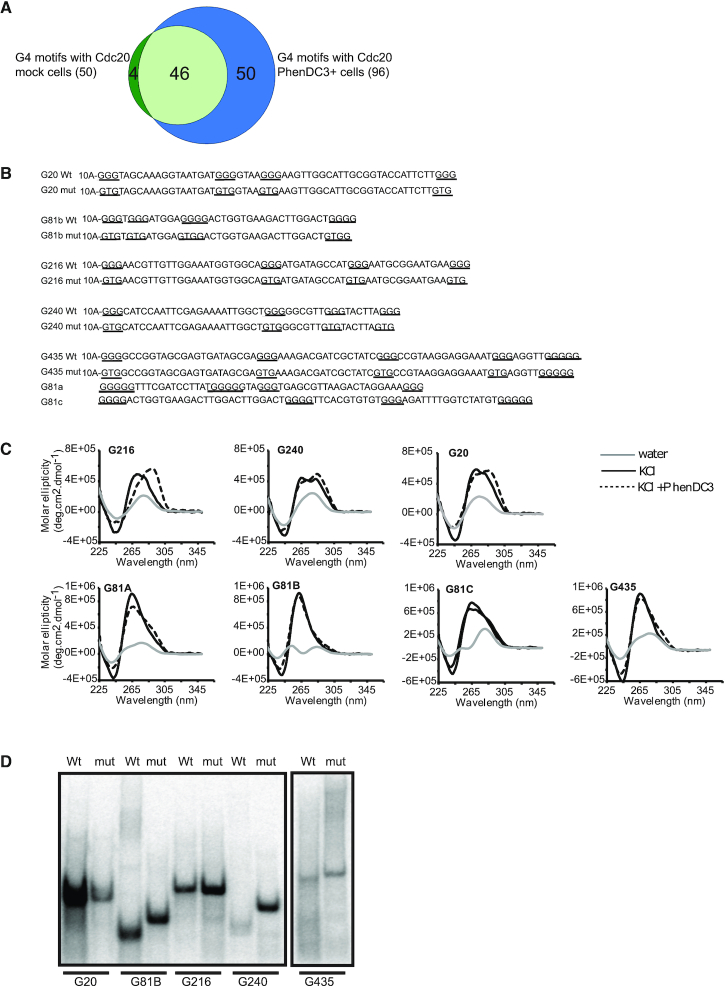
Cdc20-associated G4 motifs form G4 structures *in vitro*. (**A**) The Venn diagram shows the overlaps between G4 motifs associated with high Cdc20 occupancy in the absence and presence of 20 μM PhenDC3. The numbers in parentheses show the total number of G4 motifs in the two ChIP-seq conditions. (**B**) Sequences of the oligonucleotides used in the *in vitro* studies. The G4 motifs and their corresponding oligonucleotides were numbered based on their position in the genome (Supporting File 2). The oligonucleotides for the CD in (C) did not include 5′ poly A tails. The non-mutated and mutated G-tracts are underlined. (**C**) CD spectra of folded G216, G240, G435, G81A, G81B, G81C and G20 oligonucleotides. The G81 sequence is 123 nt long and was therefore truncated into G81A, G81B and G81C. The G4 oligonucleotides were folded in water, 100 mM KCl with 10 μM PhenDC3, or DMSO, and the CD spectra were recorded between 225 and 350 nm. OriginLab 2015 was used to smoothen the curves. The gray, black and dotted lines show G4s folded in water, KCl or KCl-PhenDC3, respectively. (**D**) Native gel electrophoresis was used to probe the molecularity of G216, G240, G435, G81b and G20. Radiolabeled oligonucleotides of the G4s (wt) or their mutated variants (mut) were folded in the presence of KCl. The folded oligonucleotides were run on 10% polyacrylamide gels containing 50 mM KCl.

Each ChIP-seq experiment (PhenDC3-treated and untreated) was performed in duplicate. A high Pearson correlation coefficient between the duplicate samples indicated good concordance between the two replicates for both experimental conditions (*r* = 0.97 and *r* = 0.99 in untreated and PhenDC3-treated samples, respectively) (Table 1). Using MACS2, we identified 344 high-confidence peaks in the untreated samples and 568 high-confidence peaks in the PhenDC3-treated samples (Table 1; Supporting File 1) (*P* < 0.005). Next, we compared the overlap of these peaks with the 446 previously identified G4 motifs (Supporting File 2) (8). The number of Cdc20 peaks in the untreated and PhenDC3-treated samples that overlapped with G4 motifs were 43 and 80, respectively (Table 2). Moreover, the number of G4 motifs in untreated and PhenDC3-treated samples that overlapped with the Cdc20 peaks were 50 and 96, respectively (Table 2 and Figure 2A, Supporting File 3). Furthermore, a majority of the G4 motifs found in the untreated condition were also found in the treated condition (Figure 2A).

**Table 2.** Fold change of selected genomic features in PhenDC3-treated samples, all significant peaks

Sample	IDR	Tot bp covered	Average peak length	Peak GC content (%)	Cdc20 peaks with G4	G4 within peaks	tRNA within peaks	5S rRNA within peaks	Fold change bp covered	Fold change G4	Fold change tRNA	Fold change 5S rRNA
mock	344	464876	1351	41.2	43	50	44	7	1.62	1.92	1.32	2.29
treated	568	752369	1325	41.6	80	96	58	16				

Because the number of peaks detected under specific conditions is not only dependent on the treatment of the sample, but also sensitive to the quality of each immunoprecipitation, we also determined the ratio of total base pairs covered by the peaks between the two conditions (752369/464876 = 1.62) (Table 2), and compared it to the fold change (96/50 = 1.92) of G4 motifs that overlapped with Cdc20 peaks for both treated (96 G4 motifs) and untreated (50 G4 motifs) samples (Table 2). While the number of bases covered by a Cdc20 peak increased 1.62 fold between untreated and PhenDC3-treated samples, the ratio for G4s that overlapped with Cdc20 peaks increased 1.92-fold (Table 2), suggesting that PhenDC3 treatment increases the overall association of Cdc20 to the genome and that this is particularly enhanced at G4 motifs. Two genomic features known to cause slow movement of the replication fork, and hence Cdc20 enrichment, are transfer DNA (tDNA) and 5S rDNA genes (63). Thus, if PhenDC3 selectively binds G4 structures, then these sites should not be significantly affected by PhenDC3 treatment. In *S. pombe*, both tDNA and 5S rDNA genes are dispersed throughout the genome (64). The fold change (58/44 = 1.32) of tDNA genes covered by the peaks in the treated and untreated samples did not increase as much as the ratio of bases covered by the Cdc20 peaks in the treated and untreated samples (fold change: 752369/464876 = 1.62), suggesting that PhenDC3 treatment does not affect Cdc20 association to tDNA genes. However, and unexpectedly, the fold change (16/7 = 2.29) of 5S rDNA genes covered by peaks in treated cells was higher than the ratio for covered base pairs (2.29 versus 1.62), suggesting that PhenDC3 treatment affected the Cdc20 association to these sites. Surprisingly, this fold change was higher for the 5S rDNA genes than for the G4 motifs (2.29 versus 1.92) (Table 2).

Next, we performed a more stringent discrimination of the identified peaks and focused on the 100 most-consistent Cdc20 peaks in the untreated and treated samples according to IDR (Supporting File 1). The number of Cdc20 peaks in the untreated and PhenDC3-treated samples that overlapped with G4 motifs were 13 and 22, respectively (Table 2), and the number of G4 motifs in untreated and PhenDC3-treated samples within each corresponding Cdc20 peaks was 16 and 27, respectively. Furthermore, we still detected a greater fold increase of G4 motifs (27/16 = 1.69) in treated samples compared to the fold increase of bases covered (187590/141280 = 1.33). However, both tDNA (18/22 = 0.82) and 5S rDNA (6/5 = 1.2) genes did not increase as much as the number of bases covered by the peaks (ratio 1.33; Table 3). This indicates a specific increase of predicted G4 structures covered by a peak after PhenDC3 treatment for the 100 most consistent Cdc20 peaks found by MACS2. Taken together, these ChIP-seq results indicate that PhenDC3 affects the stability of the predicted G4 structures, resulting in a higher number of G4 motifs within Cdc20 peaks after PhenDC3 treatment, and that the most valid G4 motifs may be the ones found in the 100 most consistent peaks. However, PhenDC3 treatment also slowed down replication of other sites, such as 5S rDNA genes, which was a surprising finding.

**Table 3.** Fold change of selected genomic features in PhenDC3-treated sample, the 100 most consistent peaks

Sample	IDR	Tot bp covered	Average peak length	Peak GC content (%)	Cdc20 peaks with G4	G4 within peaks	tRNA within peaks	5S rRNA within peaks	Fold change bp covered	Fold change G4	Fold change tRNA	Fold change 5S rRNA
mock	100	141280	1413	40.1	13	16	22	5	1.33	1.69	0.82	1.2
treated	100	187590	1876	40.7	22	27	18	6				

### *In vitro* validation of G4 motifs

We selected a subset of the G4 motifs (G20, G216, G240 and G435) identified in both treated and untreated ChIP-seq analyses that were not close to any tDNA or 5S rDNA genes, and performed several different *in vitro* analyses to determine if these sites could form G4 structures *in vitro* (Figure 2B). Twelve nucleotides or less is a common loop length limit in many bioinformatics studies for DNA sequences that potentially fold into intramolecular G4 structures (7,65,66). However, both in this study and earlier studies we used an algorithm that includes loop lengths of 1–25 nucleotides (8). To determine if the G4 motifs overlapped with a Cdc20 ChIP-seq peak, we selected four G4 motifs that contained at least one loop region that was longer than 12 nucleotides. As another test site, we also selected one G4 motif (G81) that was not Cdc20-associated in our ChIP-seq data but that contained long loops to match the four selected G4 motifs.

We first used circular dichroism (CD), which is a versatile and common technique for studying G4 structure formation and topology (67–69). DNA oligonucleotides with the G4 motif sequences were first folded either in KCl or water (control), and then PhenDC3 or DMSO was added to the folded oligonucleotides (Figure 2B). As depicted in Figure 2C, the CD spectrum of G216 in the presence of KCl + DMSO showed a positive peak at ∼260 nm, which is a characteristic spectrum of a parallel G4 structure, while the addition of PhenDC3 to the folded G216 oligonucleotide shifted the peak to ∼290 nm, indicating that PhenDC3 transformed the parallel topology to an anti-parallel G4 topology. G435 also formed a parallel G4 structure (Figure 2C), but unlike G216 the presence of PhenDC3 did not induce a significant structural change. Both G240 and G20 formed hybrid structures, with a double peak at ∼260 and ∼290 nm, and the presence of PhenDC3 slightly affected these spectra by shifting them toward the anti-parallel topology (Figure 2C). The long G81 sequence was truncated into three sequences, G81A, G81B and G81C, and all three showed parallel G4 topologies that were only slightly affected by the presence of PhenDC3 (Figure 2C). Finally, neither of the oligonucleotides folded in water showed typical G4 spectra, showing that KCl is needed to form stable G4 structures (Figure 2C). Together, the CD analysis showed that all five G4 motifs formed G4 structures *in vitro*.

Next, we used native polyacrylamide gel electrophoresis (PAGE) to determine if the oligonucleotides formed intra- or intermolecular G4 structures. On a native gel, an intramolecular G4 structure that forms within a DNA molecule migrates faster due to its compact nature compared to its ssDNA counterpart, while an intermolecular G4 structure formed between two or more DNA molecules migrates slower due to its larger size (70,71). Because we used KCl when running the native PAGE gels, we used oligonucleotides where we switched the second guanine to a thymine in each of the four G-tracts as ssDNA controls instead of the unfolded G4 oligonucleotides. These mutations most likely disrupt/destabilize the G4 structure. Wt oligonucleotides of G81B, G240 and G435 folded in KCl displayed faster migration on the native polyacrylamide gel than their mutated counterparts, indicating that these folded into a compact intramolecular G4 structure (Figure 2D), while the wt oligonucleotides of G20 and G216 folded with KCl migrated at the same rate as their mutated single-stranded counterparts, suggesting that these oligonucleotides either do not form G4 structures or form less stable G4 structures that become destabilized while running through the gel (Figure 2D). Together, these results show that under the conditions used for the native gels, neither of the oligonucleotides formed intermolecular G4 structures and most likely formed intramolecular G4 structures as predicted by the algorithm that was used.

### DNA containing the selected G4 motifs stalls the DNA polymerase one nucleotide before the first G-tract

**Figure 3.**
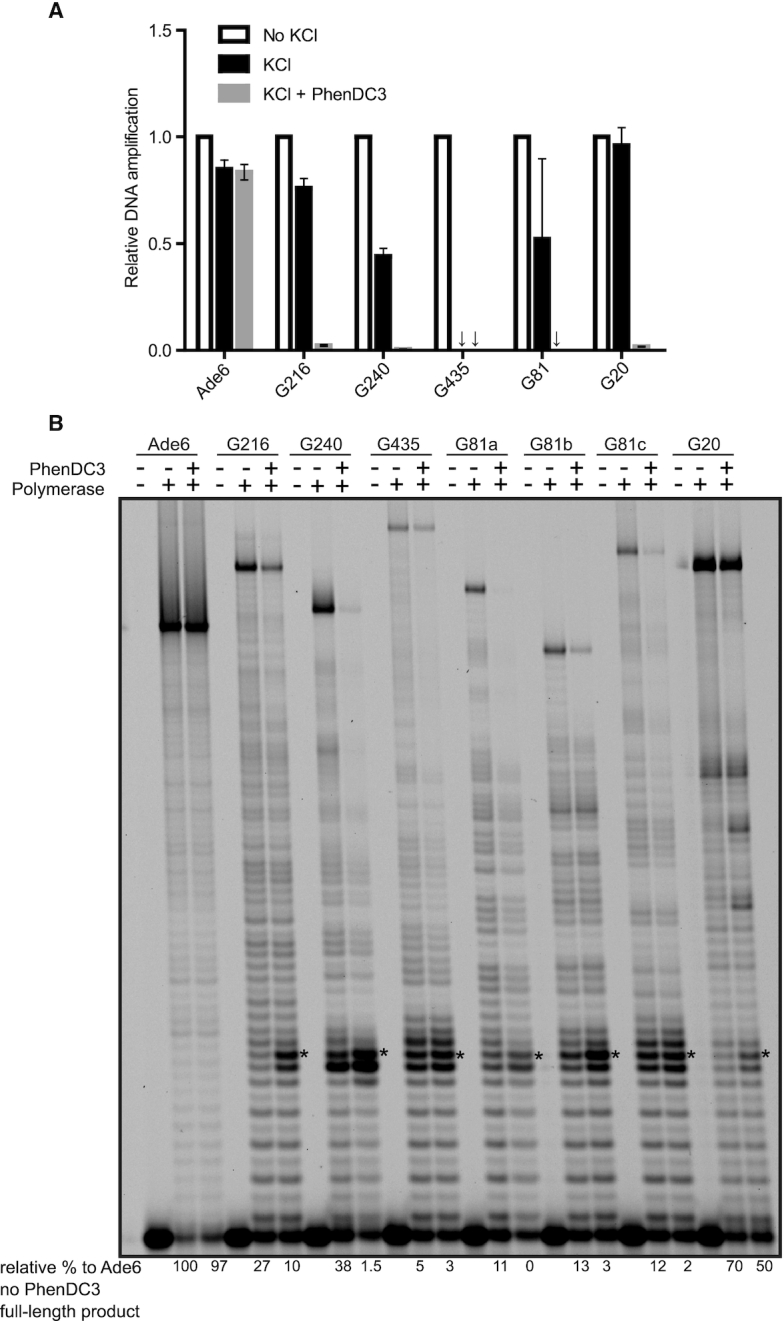
*In vitro* inhibition of DNA synthesis at the selected G4 sites. (**A**) The qPCR stop assay was performed either in the absence of KCl or in the presence of 25 mM KCl with or without 0.5 μM PhenDC3. Isolated *S. pombe* genomic DNA was used as the DNA template, and the primer pairs were designed to flank the G4 or non-G4 (*ade6^+^*) sites. The graph shows the average values of two independent experiments, with both experiments performed in technical triplicates. Error bars represent the standard deviation. (**B**) A primer extension assay was performed with different oligonucleotide templates including the sequences of G216, G240, G435, G81, G20 or non-G4 (*S. pombe ade6* gene). * indicates the first G in the first G-tract of the G4 structures. The numbers on the bottom of the gel indicate the relative percentage of amplified products relative to the untreated non-G4 control (*ade6*).

Taken together, our *in vitro* and *in vivo* data suggest that the tested G4 motifs form G4 structures and that replication is slowed down in the presence of PhenDC3. To determine if the tested G4 structures slow down replication *in vitro*, we utilized the qPCR stop assay (42). For these experiments, we isolated *S. pombe* genomic DNA and ran the qPCR in the presence or absence of PhenDC3 and KCl using primer pairs that annealed specifically to a region covering each of the five G4 motifs. If the G4 motif in this genomic region were to form a G4 structure, it would inhibit DNA replication, and therefore the amount of amplified DNA would be reduced in the presence of KCl and/or PhenDC3. In the presence of only KCl, the synthesis of genomic regions containing G81, G240 and G435 was clearly inhibited (Figure 3A). The addition of PhenDC3 resulted in complete inhibition of amplification of all five G4 structures in these genomic regions, suggesting that stabilization of these G4 structures stalled replication at these sites. In contrast, a non-G4 region (*ade6^+^*) sequence showed almost no inhibition of amplification of its DNA under any of the conditions (Figure 3A). These results suggest that all five selected G4 motifs form obstacles to DNA replication *in vitro*.

To study the replication of these sites with single-nucleotide resolution, we performed DNA primer extension assays (72). For these experiments, we annealed a primer to templates containing the selected G4 motifs and the non-G4 *ade6^+^* sequence and performed the experiments with the exonuclease-deficient Klenow fragment that is active at 37°C. Extension of the primer-templates was measured in the presence of KCl or a mixture of KCl and PhenDC3. Due to its length, G81 was again truncated into three G4 motif-containing oligos (G81A, G81B and G81C). Compared to the non-G4 DNA, the templates containing G4 motifs showed reduced amounts of full-length products in the presence of KCl (Figure 3B). The addition of PhenDC3 increased the inhibition of DNA extension for all five G4 motifs. Furthermore, clear stalling was seen one nucleotide before the start of the first G-tract in the G4 motifs (Figure 3B). Finally, as observed with the qPCR stop assay, G4 structures formed by G240, G435 and G81 truncations (G81A, G81B and G81C) showed the highest inhibitory effect on DNA amplification, indicating that these structures are the most stable G4 structures among those tested in this study.

### Recombinant Pfh1 unwinds the selected intramolecular G4 structures

In *S. pombe*, Pfh1 is needed to promote DNA replication past predicted G4 structures (46). We therefore performed helicase assays with two of the stable G4 structures, G240 and G81B, to determine whether Pfh1 can unwind the intramolecular G4 structures. The faster migrating band shifted upward, showing that Pfh1 unwound the G4 structure formed by G240 into ssDNA in a protein concentration-dependent manner (lanes 3–6 in Supplementary Figure S3A), and quantification of the gel bands showed that >50% of the 1 nM intramolecular G4 substrate was unwound to ssDNA by 9 nM Pfh1 (Supplementary Figure S3B).

**Figure 4.**
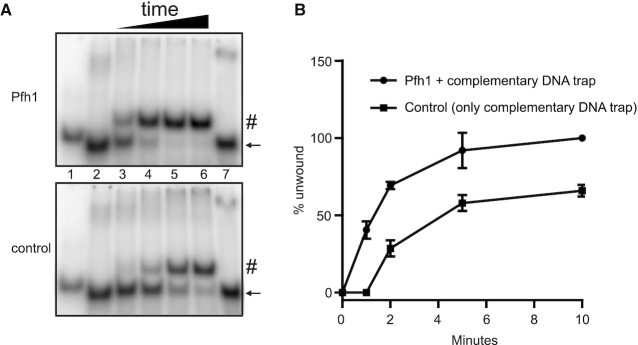
Pfh1 unwinds an intramolecular G4 structure. (**A**) The helicase-trap assay was performed using folded G81B oligonucleotide in the presence of 9 nM Pfh1 and oligonucleotide complementary to the sequence of G81B. The reaction products were separated on 10% native polyacrylamide gels containing 50 mM KCl. Lane 1: only ssDNA (mutated G81B), lane 2: folded G81B without protein, lanes 3–6: folded G81B with Pfh1 (upper gel) or BSA (lower gel) at 1, 2, 5 and 10 min, lane 7: folded G81B with helicase stop buffer. The black arrows and # indicate folded G81B and resolved G4 structures, respectively. (**B**) Quantification of the unwound G81B substrate showing the mean percentage of unwound G81B substrate from two independent experiments. Error bars represent the absolute errors.

Surprisingly, performing the helicase assay with Pfh1 to unwind the G4 structure formed by G81B showed no upward shifted band (unwound G4) (Supplementary Figure S3C). This could be a result of refolding of the unwound G81B oligonucleotide into a G4 structure because the gel contained KCl. To avoid refolding of the unwound G4 structure, we included a cold complementary DNA trap in the helicase assay, which when hybridized with the G4 motif resulted in a slower migrating band compared to the ssDNA band. Although the presence of the complementary trap DNA induced G4 disruption, the presence of Pfh1 enhanced the kinetics of G4 unwinding (lanes 3–6 in Figure 4A and B). After reacting for 2 min, 70% of the intramolecular G4 substrate was unwound to ssDNA by 9 nM Pfh1 compared to about 25% in the absence of Pfh1 (Figure 4). Thus, Pfh1 also has the ability to unwind the G81B G4 structure; however, due to fast refolding of the G4 structure, unfolding by Pfh1 was not detected in the absence of a complementary trap oligonucleotide. Together, these results suggest that Pfh1 can resolve these stable intramolecular G4 structures.

### PhenDC3 induces ssDNA lesions in *S. pombe*

**Figure 5.**
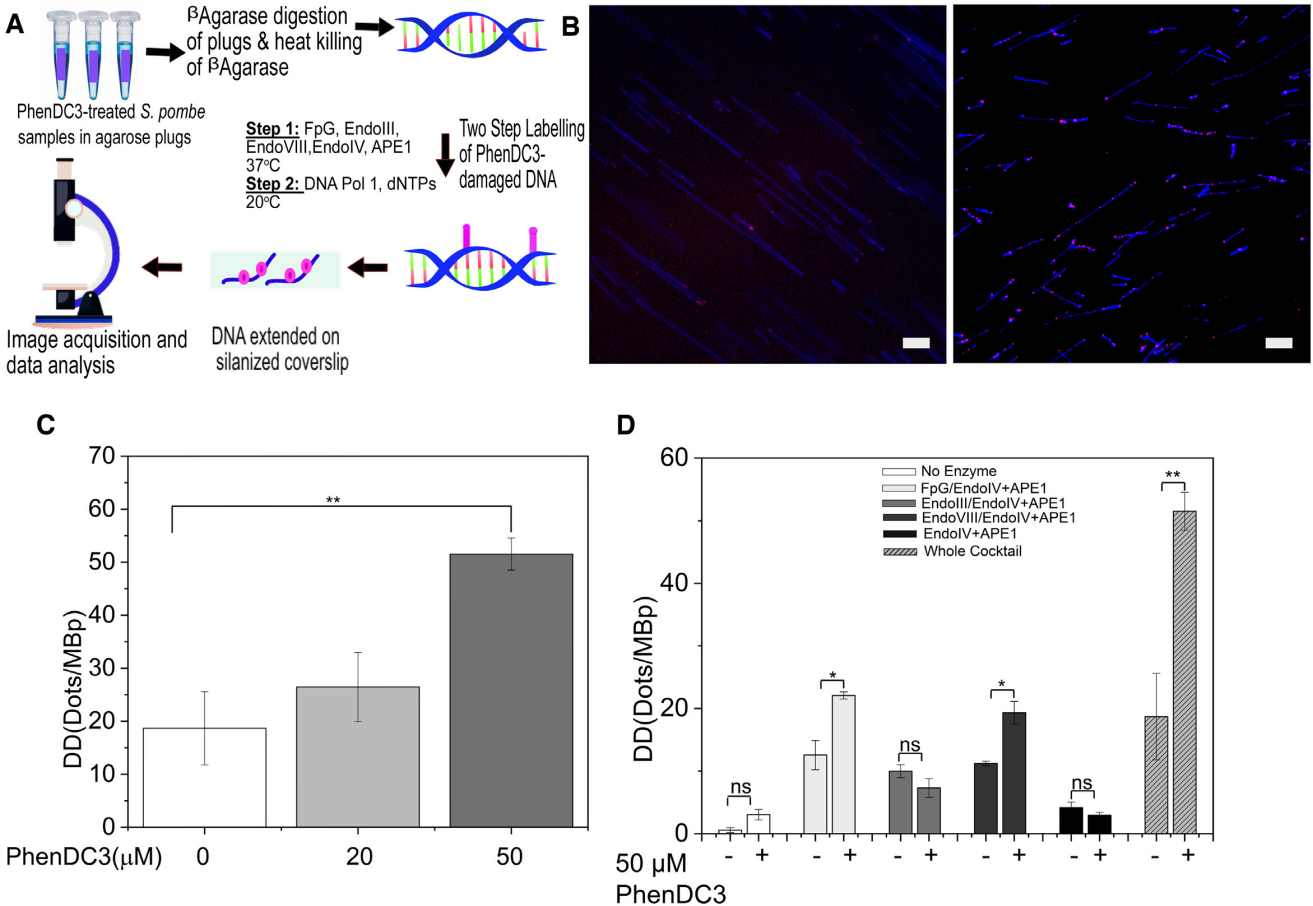
PhenDC3 induces ssDNA lesions. (**A**) Scheme of the sample preparation for PhenDC3-induced DNA damage. Labeling of damaged sites was performed by treating DNA isolated from untreated/PhenDC3-treated *S. pombe* cells with a combination of base excision repair enzymes (the enzyme cocktail) to initiate the removal of damaged DNA bases followed by gap filling with DNA pol 1 and dNTPs. (**B**) Representative microscopic images of DNA isolated from untreated (left) and 50 μM PhenDC3-treated (right) *S. pombe* cells. The DNA backbone was stained with YOYO-1 (blue), and the lesions were labeled with aminoallyl-dUTP-ATTO-647N (magenta); scale bar = 10 μm. (**C**) DNA damage in *S. pombe* exposed to increasing concentrations of PhenDC3 using the whole enzyme cocktail. Standard deviations were calculated from biological triplicates. ** *P* < 0.01 according to Student’s *t*test. (**D**) DNA damage in untreated/PhenDC3-treated *S. pombe* cells labeled using five enzyme combinations and one as no enzyme control. Standard deviations were calculated from biological duplicates. * *P* < 0.05 and ** *P* < 0.01 according to Student’s *t*test; ns = non-significant.

ChIP-seq analysis showed that cells depleted of Pfh1 not only experience replication fork stalling, but also increased DNA damage as detected by γ-H2A, a marker of DNA damage (46). To determine if G4 stabilization by PhenDC3 causes ssDNA damage in *S. pombe*, we measured the formation of DNA lesions in PhenDC3-treated *MDRΔ* cells using a DNA damage detection assay based on imaging of single DNA molecules (43,44). In this DNA damage detection assay, we labeled the ssDNA breaks formed in genomic DNA isolated from PhenDC3-treated and untreated *MDRΔ* (YJJ21) cells (Figure 5A). First, an BER enzyme cocktail containing glycosylases and endonucleases (Table 4) was used to process the DNA damage for repair, and in the second step gap filling was performed by DNA pol 1 in the presence of unlabeled dNTPs and fluorescently labeled aminoallyl-dUTP-ATTO-647N (Figure 5A). To visualize the damage sites we stretched the DNA, stained with YOYO-1, on silanized coverslips. If PhenDC3 treatment induced ssDNA damage, we would expect increased detected damage (DD) as visualized by fluorescent ‘dots’ along the single DNA molecules. Indeed, we found that DD increased approximately 3-fold with 50 μM PhenDC3 treatment compared to DMSO-treated control samples when using the whole enzyme cocktail (Figure 5B and C), suggesting that these cells have increased levels of ssDNA lesions. In cells treated with 20 μM PhenDC3, we detected a small, but not statistically significant, increase in DD, which was in line with the cell growth assays that did not show growth defects at 20 μM PhenDC3 (Supplementary Figure S2).

**Table 4.** Function of the enzymes used in the base excision repair enzyme mixture for initiating *in vitro* repair of the PhenDC3-induced DNA damage

Repair Enzyme	Function of repair enzyme
Formamidopyrimidine DNA glycosylase	Recognizes and removes 8-oxo-7,8- dihydroguanine, 2,6-diamino-4-hydroxy-5-formamidopyrimidine
Endonuclease III	Recognizes and removes various pyrimidine lesions, that includes 5,6-dihydroxythymine, uracil glycol, 5-hydroxy-6-hydrothymine
Endonuclease IV	Recognizes and acts on abasic sites, 3′-phosphate, 3′-phosphoglycolate
Endonuclease VIII	Recognizes and removes various pyrimidine lesions that includes urea, uracil glycol, 5,6-dihydroxythymine
Apurinic/apyrimidinic endonuclease 1	Recognizes and acts on abasic sites, 3-phosphate, 3′-phosphoglycolate

To determine what type of DNA lesions that were induced by PhenDC3, we investigated which particular enzyme combination in the cocktail that was the most important for repairing the lesions caused by 50 μM PhenDC3 (Figure 5D). Depending on the type of base lesion, BER is initiated by different DNA glycosylases. FpG initiates repair of 8-oxo-7,8- dihydroguanines and 2,6-diamino-4-hydroxy-5-formamidopyrimidines, while Endo IV and APE1 are essential for repair of AP sites, 3′-phosphates and 3′-phosphoglycolates (73,74). Endo III and Endo VIII act on common DNA substrates like thymine glycol, uracil glycol, but Endo VIII generates a 3′ phosphate end after processing the DNA lesions, which is not the case with Endo III (75). In these experiments, we performed the first step of labeling with five different enzyme combinations. All of the enzyme combinations, except the no-enzyme control, included EndoIV and APE1 (76–78). No significant DD was seen when performing the no-enzyme control, suggesting that DNA pol 1 alone is unable to label unprocessed PhenDC3-induced DNA lesions (Figure 5D). Both FpG and Endo VIII were able to repair the lesions caused by PhenDC3, as detected by an approximately 2-fold increased DD (Figure 5D). On the other hand, neither Endo III, Endo IV nor APE1 alone were able to process PhenDC3-induced DNA damage, suggesting that these endonucleases do not play important roles in processing DNA lesions caused by PhenDC3 (Figure 5D). Endo IV and APE1 are, however, needed to process the sites of damage after the action of FpG or Endo VIII (Figure 5D). Together, these results show that PhenDC3 causes complex types of DNA damage that can induce ssDNA breaks in *S. pombe* cells.

## DISCUSSION

Here, we have used a range of approaches to study the formation of G4 structures in *S. pombe*, a fission yeast species that diverged from the budding yeast *S. cerevisiae* >1 billion years ago (79). By using two different types of single molecule approaches, we could track individual DNA molecules and show that the well-known G4 stabilizer PhenDC3 impedes DNA replication in *S. pombe* and causes ssDNA lesions. Furthermore, several additional *in vivo* and *in vitro* experiments showed the folding and unfolding of G4 structures at G4 motifs, which together indicate that G4 structures form in the *S. pombe* genome.

Flow cytometry analysis demonstrated that high concentrations of PhenDC3 caused a prolonged S-phase, indicating that formation of G4 structures is replication-dependent. S-phase-dependent G4 formation is also detected in fixed human cells by the G4-specific antibody, BG4 (80), showing that not only the location of G4 motifs in the genome is evolutionary conserved between these two organisms (7,9,46), but also the cell cycle phase at which they form is conserved. At a lower PhenDC3 concentration, the rate of DNA synthesis was slowed down in the presence of PhenDC3; however, the *S. pombe* cells completed S-phase without any delays, perhaps by firing late or dormant origins of replication (81,82). Indeed, we observed greater numbers of replication tracts in PhenDC3-treated cells compared to the non-treated cells, suggesting an increased number of DNA replication origin firings. Furthermore, we detected increased levels of Cdc20, the catalytic subunit of Polϵ, in the PhenDC3-treated cells. Polϵ not only participates in leading-strand DNA synthesis (83,84), but is also necessary for the assembly of the replisome for replication initiation (62,85). The increased levels of Cdc20 that we observed might therefore be due to the activation of dormant origins and therefore to a greater number of assembled replisomes to compensate for the slower forks or might be because there are more paused Cdc20 molecules due to stable G4 sites, or a combination thereof. Because we did not observe increased Pfh1 protein levels, and because Pfh1 is also a replisome component (86), the increased Cdc20 protein levels are more likely to be due to the activation of late origins in PhenDC3-treated cells. Similar to our observations in *S. pombe*, mouse embryonic stem cells treated with low concentrations of PhenDC3 do not demonstrate any defects in cell cycle progression and do not activate cell cycle checkpoints, although PhenDC3 treatment results in the firing of a new set of origins (87).

Moreover, the single DNA molecule damage detection assay support the *in vivo* results, showing that 50 μM PhenDC3 is deleterious for the cells and suggesting that at lower PhenDC3 concentrations the cells have the capacity to resolve and/or repair the PhenDC3-induced lesions. DNA pol 1 alone was unable to label the ssDNA lesions caused by PhenDC3, suggesting that the PhenDC3-induced damages were complex in nature. In *E. coli*, oxidative pyrimidine lesions are repaired by endonucleases like Endo III and Endo VIII (88,89). However, Endo III was unable to process the PhenDC3-induced DNA lesions, while Endo VIII was important for removing the damaged bases resulting from PhenDC3 treatment. This agrees with the observation that, NEIL1, the human Endo VIII homolog, is more efficient at removing damaged bases from G4 DNA when compared to NTH1, the human Endo III homolog (90). Also, 3′-end proximal oxidative DNA lesions resistant to NTH1 can be cleaved by NEIL1 (91). The inability of Endo IV and APE1 to initiate repair of the DNA lesions formed by PhenDC3 stabilization of G4 structures suggests that abasic sites were not a major DNA lesion. G-rich telomeres can accumulate oxidized guanine species, such as 8-oxo-7,8-dihydroguanine, and FpG recognizes and specifically removes these types of DNA lesions (92,93). We demonstrated that FpG was able to repair the damages detected in the PhenDC3-treated cells, thus there is the possibility that accumulation of 8-oxo-7,8-dihydroguanine lesions in PhenDC3-treated samples might not have been repaired within the cells due to constraints induced by the stabilized G4 structures. In fact, DNA end resection during homologous recombination is impaired in pyridostatin-treated human cells, suggesting that stabilized G4 structures hinder the processing of DNA ends by the homologous recombination machinery (94).

High occupancy by *S. cerevisiae* DNA Pol2 and *S. pombe* Cdc20 are used to identify sites where replication progresses slowly in the genome (8,95). In the 100 most consistent Cdc20 peaks, we detected an increased number of predicted G4 structures in PhenDC3-treated cells compared to the untreated cells, suggesting that PhenDC3 stabilization slows down replication past these G4 structures and that less-stable G4 structures can be detected through further stabilization by the ligand. We focused these studies on G4 motifs comprised of four repeats of three guanines separated by up to 25 nucleotides, an algorithm which have been used for predicting G4 structures that form three stacks of G-tetrads in budding and fission yeast species (5,46). However, the prediction of sequences that can form G4 structures is still being refined by new algorithms, newly developed methods, and experimental analyses (47,96). For example, using G4-sequencing, 700 000 G4 structures were predicted in human cells; however, almost 50% of these G4 motifs were not canonical G4 sequences and included longer loops and bulges within the G4 structures (18). Also, two-tetrad G4 structures can form *in vitro* (97). Therefore, the number of predicted G4 structures that we identify will likely change depending on which algorithm we use to perform our analysis. Although 5S rRNA encoding genes do not overlap with motifs that are predicted to form three stack-layered G4 structures (46), we found that all *S. pombe* 5S rDNA genes contain at least one predicted two stack-layered G4 structure on the non-transcribed strand (see Supporting File 2 for sequences). This can explain our surprising findings that PhenDC3-treated cells showed enhanced Cdc20-association to 5S rDNA genes and suggests that PhenDC3 binds and stabilizes these two stack-layered G4 structures, which results in slower replication fork movement. Formation of both canonical and non-canonical RNA G4 structures in human cells are reported, and these structures are implicated in either transcriptional or translational regulation (98,99). Because the *S. pombe* G4 motifs are located in the non-transcribed strands of 5S rDNA genes, the formation of G4 structures may have a function in the rRNA. In human cells, non-canonical structures in the 5S rRNA are suggested as binding sites for proteins that facilitate the transport of 5S rRNA molecules into the mitochondria (100). However, transport of 5S rRNA molecules in the mitochondria of *S. pombe* cells is still not yet reported.

We used four complementary *in vitro* assays and demonstrated that the selected G4 sequences form G4 structures, and that several of the oligonucleotides formed intramolecular parallel G4 structures. Intramolecular G4 topologies will be the most frequent and plausible forms of G4 structures in the cells, especially in cases where the G4 loci are found as single copies, and formation of parallel topologies are favored (4,42). Both the polymerase stop assay and the qPCR stop assay suggested that G20 and G216 were less stable than G81, G240, and G435, but it was evident that PhenDC3 increased the stability of all five G4 structures. Based on both replication assays, and the fact that PhenDC3 was able to change the structure of G20 and G216 according to the CD analysis, we rank the stability of the formed G4 structures in the order G435 > G240 > G81 > G216 > G20. However, this ranking might not correspond with the stability of the G4 structures *in vivo* because the stability of G4 structures is also influenced by molecular crowding conditions, negative supercoiling and the chromatin status of the investigated DNA regions (101–103). Furthermore, at least one of the loop regions in each G4 motif tested had a loop region longer than 12 nucleotides, and in these cases the long loop region did not seem to obviously influence the formation and stability of these G4 structures. In fact, G435, which we ranked as the most stable among the five tested G4 structures, had three long loop regions. *In vitro*, G4 sequences from the *S. cerevisiae* genome can also form G4 structures from a broad range of loop sizes and sequences (4).

Pfh1 is so far the only known G4 unwinder in *S. pombe*, and it has been shown that Pfh1 can unwind G4 structures formed in both rDNA and telomeric G4 sequences (27). We demonstrated that recombinant and purified nuclear Pfh1 (27) unwound the tested G4 sequences that formed either parallel or hybrid intramolecular G4 structures, suggesting that Pfh1 processes structures with different G4 topologies, although, the kinetics of unwinding may differ dependent on the stability of the structure (4,28,104). Our data indicated that 8-oxo-7,8-dihydroguanine lesions in PhenDC3-treated samples may not have been repaired. *In vitro*, Pfh1 can unwind PhenDC3-stabilized G4 structures, however at lower rate than non-stabilized G4 structures (27). Association and unwinding of 8-oxo-7,8-dihydroguanine-containing G4 structures by Pfh1 has not yet been studied, but one possibility can be that these G4 structures are not recognized and unwound by Pfh1, or that the rate of unwinding is too slow, which results in ssDNA breaks. The role of Pif1 helicases in cells might not only involve promoting G4 unwinding during DNA replication, but an additional conserved role of Pif1 helicases might be to suppress genomic rearrangements at G4 structures (21), perhaps by promoting resection at G4 structures that are induced by DNA double-strand breaks (21,94).

In summary, we show that stabilized G4 structures are impediments for DNA replication and that G4 stabilization can cause ssDNA lesions, suggesting that unresolved G4 structures are natural replication barriers. Our results provide a framework for further investigation of the biological functions of G4 structures in *S. pombe* that will enhance our understanding of the roles that G4 structures play in humans and in the onset of human diseases connected to G4 structures.

## DATA AVAILABILITY

The sequencing data have been deposited at the European Nucleotide Archive (ENA, www.ebi.ac.uk/ena) under accession number PRJEB37862.

## Supplementary Material

gkaa820_Supplemental_Files
