# Supplementary material for: Stabilization of G-quadruplex DNA structures in Schizosaccharomyces pombe causes single-strand DNA lesions and impedes DNA replication
Source: Nucleic Acids Res. 2020 Oct 12;48(19):10998–1015. doi: 10.1093/nar/gkaa820 (PMC7641769; doi:10.1093/nar/gkaa820)
Supplement: gkaa820_Supplemental_Files [file gkaa820_supplemental_files.zip › Supplementary data_revised.pdf]

Supporting Information

Stabilization of G-quadruplex DNA structures in *Schizosaccharomyces pombe* cause single-strand DNA lesions and impede DNA replication

Ikenna Obi[a], Matilda Rentoft[a], Vandana Singh[b], Jan Jamroskovic[a], Karam Chand[c], Erik Chorell[c], Fredrik Westerlund[b], and Nasim Sabouri[a#]

[a] Department of Medical Biochemistry and Biophysics, Umeå University, 901 87 Umeå, Sweden.

[b] Department of Biology and Biological Engineering, Chalmers University of Technology, 412 96 Gothenburg, Sweden.

[c] Department of Chemistry, Umeå University, 901 87 Umeå, Sweden

[#] corresponding author: nasim.sabouri@umu.se

Supplementary Figures

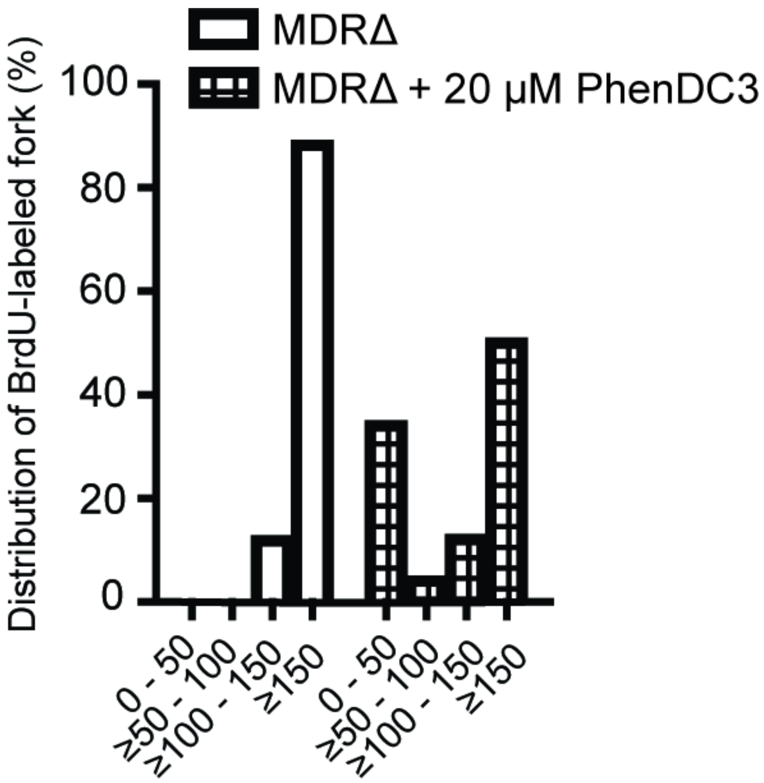

Supplementary Figure 1. The distribution frequency of BrdU-labeled forks of particular sizes from the DNA fiber analysis in Figure 1D. Lengths of DNA fibers were measured and assigned to different groups. The data show the percentage of the groups in a given sample population.

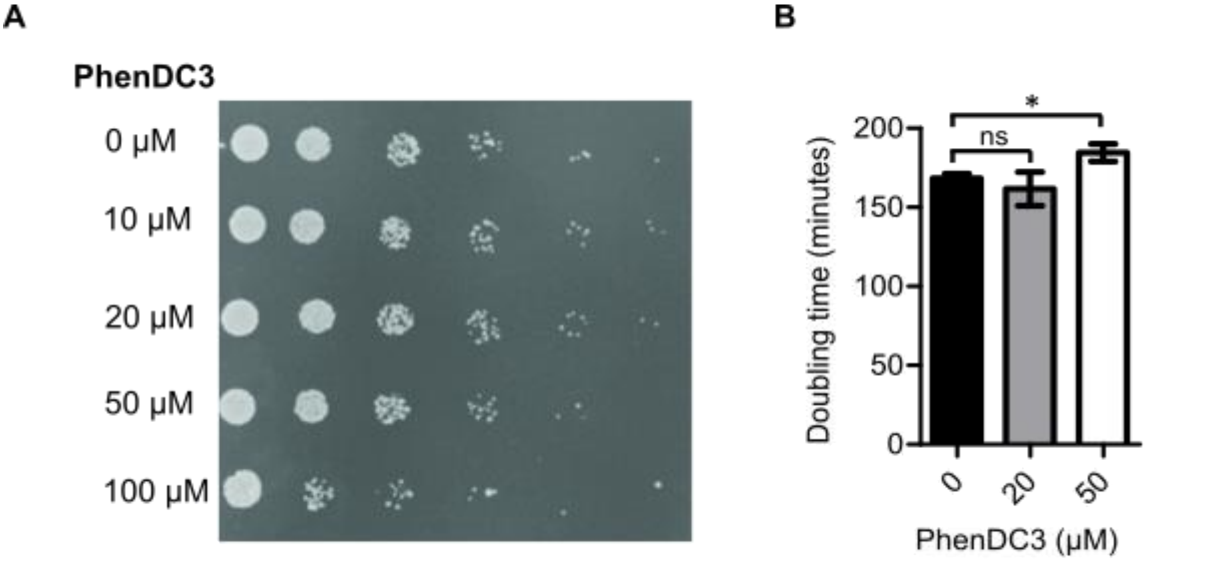

Supplementary Figure 2. Screening of optimal PhenDC3 concentrations of MDRΔ *S. pombe* growth for ChIP analysis. **(A)** A total of 1 × 10^6 *cdc20-3HA* (YJJ21) cells per mL were grown at 30°C for 12 h in EMM2 liquid media containing increasing PhenDC3 concentrations. Cells were serial-diluted and spotted on EMM2 agar plates and allowed to grow for 4 days at 30°C. **(B)** Exponentially growing YJJ21 cells were grown in EMM2 media containing 0, 20, or 50 µM PhenDC3. The doubling time was determined for the treated cultures grown for 24h at 30°C. Three independent experiments were performed. Error bars represent the standard deviation of the mean. * p=0.0022 according to the Mann–Whitney U test. ns indicates non-significant difference.

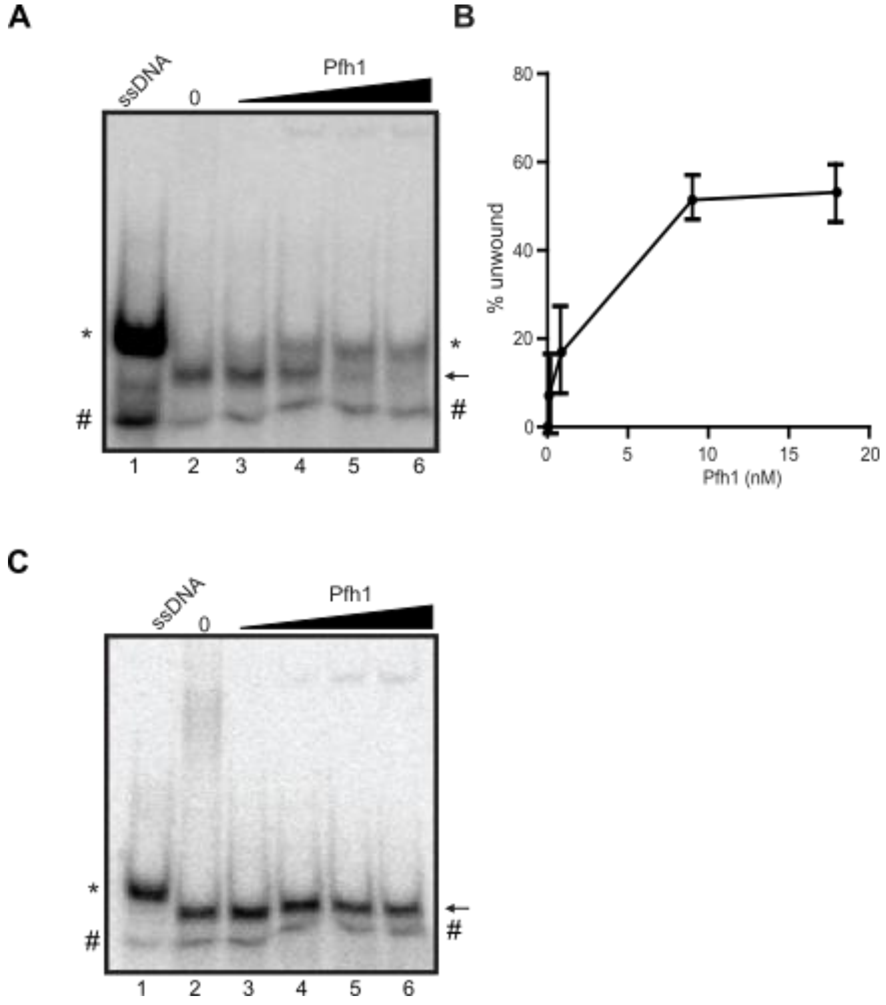

Supplementary Figure 3. Pfh1 unwinding assays. **(A)** The helicase assay was performed using 1 nM folded G240 oligonucleotide in the presence of increasing Pfh1 concentrations. Reaction products were separated on 10% native polyacrylamide gels containing 50 mM KCl. Lane 1: only ssDNA (mutated G240), lane 2: folded G240 without protein, lanes 3-6: folded G240 with 0.09, 0.9, 9, and 18 nM Pfh1. The black arrow and asterisk (*) indicate folded G240 and unwound G4 bands, respectively. # indicates free γ-ATP label. **(B)** Quantification of the unwound G240 substrate showing the mean percentage of unwound G240 substrate from two independent experiments. Error bars represent the absolute errors. **(C)** The helicase assay was performed using a folded G81B oligonucleotide in the presence of increasing Pfh1 concentrations. Reaction products were separated as described in A above. Lane 1: only ssDNA (mutated G81B), lane 2: folded G81B without protein, lanes 3–6 folded G81B with 0.09,

0.9, 9, and 18 nM Pfh1. The black arrows indicate the folded intramolecular G4 DNA. * indicates ssDNA and # indicates free γ-ATP label.

Supplementary Table 1: Yeast strains used in this study

Strain	Genotype	source
YIO4	*hbfr1::hygr pmd1::natr cdc25-22 pfh1::ura4+-nmt-pfh1-GFP, leu1-32::[hENT1 leu1+] his7-366::[hsv-tk his7+] ade6-M21? ade6-M210? his3-D1? telo-his3?*	This study
YJJ16	*bfr1::hygr pmd1::natr leu1-32::pJk148(leu)-pfh1-13MYC-kanmx6, ade6-M210?, pfh1+*	This study
YJJ21	*bfr1::hygr pmd1::natr cdc20+::cdc20-3HA-kanmx6 pfh1::ura+nmt81-pfh1-GFP leu1-32*	This study
YJJ32	*bfr1::hygr pmd::natr cdc25-22 ade6-M210 leu1-32 his3-D1?*	This study
YNS112	*leu1-32 his3-D1 ura4-D18 ade6-M210*	Pinter et al. 2008
YNS219	*ade6-M210 leu1 bfr1::hygr pmd1::natr*	Kawashima et al. 2012

Supplementary Table 2: Sequences of oligonucleotides used in this study

Oligo name	Sequence 5’-3’	Description
NS213	CAGTTTAGACGGAAAAGTTTATGC	primer for amplification of 5’ region of Ade6 (qPCR stop assay)
NS214	CACGCTGTTGAATTGAGAAGG	primer for amplification of 3’ region of Ade6 (qPCR stop assay)
IO43	GCTTTCACCGCCCTTTCTCA	primer for amplification of 5’ region of G216 (qPCR stop assay)
IO12	GGCTAGCACTAGCACTTTGC	primer for amplification of 3’ region of G216 (qPCR stop assay)
IO13	CAACGCAGGCTGTTAACTCG	primer for amplification of 5’ region of G240 (qPCR stop assay)
IO44	AAGGAGGGAGTGTCGAGAGG	primer for amplification of 3’ region of G240 (qPCR stop assay)
IO45	TCTCTCTAGGTGTGTCCTCT	primer for amplification of 5’ region of 435 (qPCR stop assay)
IO20	GCCAATAGGAGGGCGACAG	primer for amplification of 3’ region of G435 (qPCR stop assay)
IO46	TGTTAACATTGCTAATATCCTCACC	primer for amplification of 5’ region of G20 (qPCR stop assay)
IO47	CTCGTACCCTTGGTGACACT	primer for amplification of 3’ region of G20 (qPCR stop assay)
IO48	GCCACCTACGCTGACATCTT	primer for amplification of 5’ region of G81 (qPCR stop assay)
IO49	CAACCGTTGTGTTTTTGTTTTACA	primer for amplification of 3’ region of G81 (qPCR stop assay)
G216	GGGAACGTTGTTGGAAATGGTGGCAGGGATG ATAGCCATGGGAATGCGGAATGAAGGG	G216 sequence
G240	GGGCATCCAATTCGAGAAAATTGGCTGGGGG CGTTGGGTACTTAGGG	G240 sequence
G435	GGGGCCGGTAGCGAGTGATAGCGAGGGAAA GACGATCGCTATCGGGCCGTAAGGAGGAAAT GGGAGGTTGGGGG	G435 sequence
G81a	GGGGGATTTCGATCCTTATGGGGGTAGGGTG AGCGTTAAGACTAGGAAAGGG	truncated G81 sequence
G81b	GGGTGGGATGGAGGGGACTGGTGAAGACTT GGACTGGGG	truncated G81 sequence
G81c	GGGGACTGGTGAAGACTTGGACTGGGGTTC ACGTGTGTGGGAGATTTTGGTCTATGTGGGGG	truncated G81 sequence
G20	GGGTAGCAAAGGTAATGATGGGGTAAGGGAA GTTGGCATTGCGGTACCATTCTTGGG	G20 sequence

10A-G216	AAAAAAAAAAGGGAACGTTGTTGGAAATGGTGGCAGGGATGATAGCCATGGGAATGCGGAATGAAGGG	G216 sequence with 5′ poly A
10A-G240	AAAAAAAAAAGGGCATCCAATTCGAGAAAATTGGCTGGGGGCGTTGGGTACTTAGGG	G240 sequence with 5′ poly A
10A-G435	AAAAAAAAAAGGGGCCGGTAGCGAGTGATAGCGAGGGAAAGACGATCGCTATCGGGCCGTAAGGAGGAAATGGGAGGTTGGGGG	G435 sequence with 5′ poly A
10A-G81b	AAAAAAAAAAGGGTGGGATGGAGGGGACTGGTGAAGACTTGGACTGGGG	truncated G81 with 5′ poly A
10A-G20	AAAAAAAAAAGGGTAGCAAAGGTAATGATGGGGTAAGGGAAGTTGGCATTGCGGTACCATTCTTGGG	G20 sequence with 5′ poly A
10A-G216-M4	AAAAAAAAAAGTGAACGTTGTTGGAAATGGTGGCAGTGATGATAGCCATGTGAATGCGGAATGAAGTG	G216 sequence with 5′ poly A and G-tract mutations
10A-G240-M4	AAAAAAAAAAGTGCATCCAATTCGAGAAAATTGGCTGTGGGCGTTGTGTACTTAGTG	G240 sequence with 5′ poly A and G-tract mutations
10A-G435-M4	AAAAAAAAAAGTGGCCGGTAGCGAGTGATAGCGAGTGAAAGACGATCGCTATCGTGCCGTAAGGAGGAAATGTGAGGTTGGGGG	G435 sequence with 5′ poly A and G-tract mutations
10A-G81b-M4	AAAAAAAAAAGTGTGTGATGGAGTGGACTGGTGAAGACTTGGACTGTGG	truncated G81 with 5′ poly A and G-tract mutations
10A-G20-M4	AAAAAAAAAAGTGTAGCAAAGGTAATGATGTGGTAAGTGAAGTTGGCATTGCGGTACCATTCTTGTG	G20 sequence with 5′ poly A and G-tract mutations
ade6 nonG4	GAGACCATTCAAAAGGATAATGTTTGTCATTTAGTATATGCCCCTGCTCGTCTTCCCTTCTCCGGACGCTCGACGCCATTAATAATGTTTTCA	template for the polymerase stop assay for *ade6*
G216 stop	ATATATATATGGGAACGTTGTTGGAAATGGTGGCAGGGATGATAGCCATGGGAATGCGGAATGAAGGGATATATATATCGGACGCTCGACGCCATTAATAATGTTTTCA	template for the polymerase stop assay for G216
G240 stop	ATATATATATGGGCATCCAATTCGAGAAAATTGGCTGGGGGCGTTGGGTACTTAGGGATATATATATCGGACGCTCGACGCCATTAATAATGTTTTCA	template for the polymerase stop assay for G240
G435 stop	ATATATATATGGGGCCGGTAGCGAGTGATAGCGAGGGAAAGACGATCGCTATCGGGCCGTAAGGAGGAAATGGGAGGTTGGGGGATATATATATCGGACGCTCGACGCCATTAATAATGTTTTCA	template for the polymerase stop assay for G435
G81a stop	ATATATATATGGGGGATTTCGATCCTTATGGGGGTAGGGTGAGCGTTAAGACTAGGAAAGGGATATATATATCGGACGCTCGACGCCATTAATAATGTTTTCA	template for the polymerase stop assay for truncated G81
G81b stop	ATATATATATGGGTGGGATGGAGGGGACTGGTGAAGACTTGGACTGGGGATATATATATCGGACGCTCGACGCCATTAATAATGTTTTCA	template for the polymerase stop assay for truncated G81
G81c stop	ATATATATATGGGGACTGGTGAAGACTTGGACTGGGGTTCACGTGTGTGGGAGATTTTGGTCTATGTGGGGGATATATATATCGGACGCTCGACGCCATTAATAATGTTTTCA	template for the polymerase stop assay for truncated G81

G20 stop	ATATATATATGGGTAGCAAAGGTAATGATGGGGTAAGGGAAGTTGGCATTGCGGTACCATTCTTGGGATATAT<br>ATATCGGACGCTCGACGCCATTAATAATGTTTTCA	template for the polymerase stop assay for G20
rev c81b	CCCCAGTCCAAGTCTTCACCAGTCCCCTCCATCCCACCC	trap for G81b

Supplementary Table 3: Number of BrdU-labeled DNA fibers observed during the DNA fiber experiment

	MDRΔ	MDRΔ + 20µM PhenDC3
**Number of BrdU-labelled DNA (Replicate 1)**	17	50
**Number of BrdU-labelled DNA (Replicate 2)**	18	25
